# The American Institute of Homœopathy

**Published:** 1893-07

**Authors:** Pemberton Dudley

**Affiliations:** General Secretary


### THE AMERICAN INSTITUTE OF HOMŒOPATHY.

Editor Homœopathic Physician:—At the recent meeting of the Institute, the General Secretary was instructed to inform the profession, through the journals, of the important changes made in the By-Laws.

The designation "Bureau" is changed to "Section." The Bureau of Anatomy, etc., is dropped, and Pathology is included with Clinical Medicine. The "Bureau of Mental and Nervous Diseases" is to be called the "Section in Neurology." The Bureau of Organization, etc., becomes a committee. Each section must consist of at least five members; beyond that number there is no restriction. Each chairman is required to send the General Secretary, within one month after the session, the names of the officers and members of his section.

Delegates from societies and institutions will be admitted to certain privileges, as heretofore, but will not be expected to present reports. The Committee on Pharmacy is discontinued.

The session of 1894 will be held in Denver, Col. The meetings will open about the middle of the week, and extend into the next week long enough to allow each section all the time it may wish for its papers and discussions.

Pemberton Dudley, M. D.,
*General Secretary.*

